# Conservative initial postoperative anticoagulation strategy after HeartMate 3 left ventricular assist device implantation

**DOI:** 10.1007/s12471-022-01671-1

**Published:** 2022-04-05

**Authors:** Kevin Damman, Stan A. J. van den Broek, Gianclaudio Mecozzi, Joep M. Droogh, Ethel Metz, Annemieke Oude Lansink, Jan A. Krikken, Michiel E. Erasmus, Michiel Kuijpers

**Affiliations:** 1 Department of Cardiology, University Medical Center Groningen, University of Groningen, Groningen, The Netherlands; 2 Department of Cardiothoracic Surgery, University Medical Center Groningen, University of Groningen, Groningen, The Netherlands; 3 Department of Critical Care, University Medical Center Groningen, University of Groningen, Groningen, The Netherlands

**Keywords:** LVAD, HeartMate 3, Anticoagulation

## Abstract

**Introduction:**

Although anticoagulation therapy is mandated after implantation of a left ventricular assist device (LVAD), postoperative bleedings and reoperations occur relatively frequently and are associated with worse outcomes. We evaluated the use of a conservative postoperative anticoagulation protocol in patients implanted with a HeartMate 3 (HM3) LVAD.

**Methods:**

In a single-centre retrospective analysis of postoperative outcomes after HM3 LVAD implantation, a standard (old) anticoagulation protocol (i.e. early, full-dose anticoagulation with low-molecular weight heparin and overlapping vitamin K antagonist) was compared with a new conservative anticoagulation protocol (i.e. slow initiation of vitamin K antagonists without overlapping heparin). Main outcomes were changes in international normalised ratio (INR), lactate dehydrogenase (LDH), bleeding and/or tamponade events requiring reoperation, length of stay and adverse events.

**Results:**

In total, 73 patients (48 in old vs 25 in new protocol group) were evaluated. Mean age was 56 years (standard deviation 13) and most patients (78%) were males. Changes in INR and LDH in the first 14 days were similar in both groups (*p* = 0.50 and *p* = 0.997 for interaction, respectively). Number of bleeding/tamponade events requiring reoperation was lower in the new than in the old protocol group (4% vs 33%, *p* = 0.005). Postoperative 30-day mortality was similar, and we observed no thromboembolic events. Median (25th–75th percentiles) total length of postoperative hospital stay (27 (25–41) vs 21 (19–27) days, *p* < 0.001) and length of intensive care unit stay (5 (2–9) vs 2 (2–5) days, *p* = 0.022) were significantly shorter in the new protocol group.

**Conclusion:**

These retrospective data suggest that conservative slow initiation of anticoagulation therapy after HM3 LVAD implantation is associated with less bleeding/tamponade events requiring reoperation, a similar safety profile and a shorter duration of stay than the currently advised standard anticoagulation protocol.

**Supplementary Information:**

The online version of this article (10.1007/s12471-022-01671-1) contains supplementary material, which is available to authorized users.

## What’s new?

- In patients implanted with a HeartMate 3 left ventricular assist device (LVAD), immediate postoperative anticoagulation can be started conservatively.
- With this conservative strategy, the bleeding rate was significantly reduced without an increase in thromboembolic events.
- These findings further support a more conservative approach to therapeutic anticoagulation directly after implantation of a LVAD HeartMate 3 to prevent major bleeding complications.

## Introduction

Left ventricular assist device (LVAD) implantation has become an important treatment option for patients with advanced, end-stage heart failure with reduced ejection fraction (HFREF) [1–3]. The clinical outcome of patients with an LVAD has substantially improved with the introduction of newer generation devices. Most recent data have shown that with the third-generation centrifugal flow LVAD, the HeartMate 3 (HM3), the overall survival rate after 2 years is 79% and is superior to the second-generation axial flow device [3]. While thromboembolic and bleeding rates are lower than with the second-generation axial flow device, 44% of patients still experience any type of bleeding, of whom 10% require surgery [3].

Full-dose anticoagulation, together with aspirin, is still advised for the HM3 system and should be initiated early after implantation [3]. Given the limited number of thromboembolic events, it is questionable whether such a rigorous anticoagulation protocol is necessary. A small study evaluating lower INR thresholds in the chronic phase showed this is feasible and not associated with more thromboembolic events [4].

We assessed patient outcomes before and after changing to a more conservative postoperative anticoagulation protocol in patients implanted with an HM3 LVAD at our centre.

## Methods

This single-centre study retrospectively evaluated consecutive patients implanted with an HM3 LVAD from 1 November 2016 until 1 June 2020 at the University Medical Centre Groningen, the Netherlands. The clinical decision to implant an LVAD was based on the European Society of Cardiology HF guidelines and the Dutch national consensus document on LVAD therapy [1, 4, 5]. Patients were implanted with an LVAD as bridge-to-transplant, bridge-to-decision or destination therapy.

As part of quality control, inclusion of all LVAD patients in the European Registry for Patients with Mechanical Circulatory Support (EUROMACS) is mandatory in the Netherlands. Thus, all patients in our study provided written informed consent, and the data in the current manuscript reflect single-centre data from the registry [5, 6]. To ensure completeness of short-term postoperative data, including laboratory and clinical variables, an additional retrospective chart review was carried out.

### Old anticoagulation protocol

Our initial postoperative anticoagulation protocol (from here on referred to as ‘old protocol’) was used until 10 July 2019 and consisted of the following steps. Anticoagulation treatment consisting of subcutaneous full-dose low molecular-weight heparin (LMWH) adjusted to body weight was initiated when chest tube drainage < 50 ml/h over a period of 6 h, as well as < 200 ml cumulative production in the same period.

After chest tube removal and in the absence of bleeding, vitamin K antagonist treatment with acenocoumarol was initiated overlapping with full-dose LMWH. After the target INR was reached (in our centre, 1.8–2.5 for HM3 or 2.0–3.0 if history of atrial fibrillation), LMWH was discontinued. In the same period, aspirin 81–100 mg once a day (QD) was initiated and continued during LVAD support.

### New anticoagulation protocol

After reviewing the type and number of postoperative bleedings resulting in reoperation, the described postoperative anticoagulation protocol was changed on 11 July 2019 to include the following steps. On day 0 and day 1 after LVAD implantation, no anticoagulation was given. On day 2 (~ 48 h after implantation), low-dose vitamin K antagonist treatment (acenocoumarol) was initiated (start with 1 mg on day 2, based on a ‘start low, go slow’ approach to prevent high INR levels) if chest tube drainage was acceptable (similar to the old protocol). Prophylactic LMWH could be administered if the patient’s mobility was poor.

The next days, the vitamin K antagonist dose was slowly increased to reach the target INR of 1.8–2.5 mg (2.0–3.0 if history of atrial fibrillation), and care was taken to ‘go slow’ to prevent high INR levels. During the uptitration phase, no full-dose (overlapping) LMWH was administered. On day 7 or later (but not sooner), aspirin 81–100 mg QD was initiated and continued during LVAD support.

### Outcomes

We evaluated the following outcomes: (1) change in INR and percentage of patients in adequate INR range in the first 2 weeks, (2) LDH change after LVAD implantation in the first 2 weeks as a marker of haemolysis, (3) total number and time to first reoperation due to bleeding or tamponade in the first 30 days, (4) peri- and postoperative need for packed red blood cell transfusion up to 3 weeks, (5) postoperative length of stay (total and intensive care unit (ICU) time), (6) thromboembolic events and (7) postoperative mortality.

### Statistical analysis

Normally distributed continuous variables are presented as mean ± standard deviation and non-normally distributed continuous variables as median (25th–75th percentiles). Categorical variables are presented as number (percentage). Differences in baseline characteristics between the old and new anticoagulation protocol groups were evaluated using either the *t*-test, chi-square or Mann-Whitney U test, where appropriate.

Absolute levels of INR and LDH over time and their changes were investigated by repeated measures analysis mixed effect modelling using unstructured covariance. Covariates that were used as fixed effects included type of anticoagulation protocol, days after LVAD implantation and anticoagulation protocol × days after LVAD implantation interaction, with random intercept and slope on individual patient level. Time was modelled linearly.

Differences in length of stay were evaluated with the Mann-Whitney U test, while differences in event rates were evaluated with the chi-square test. The association between the old versus new anticoagulation protocol and time to first bleeding event resulting in reoperation and mortality was analysed using the Cox proportional hazard analysis and is visually depicted herein by Kaplan-Meier curves.

Two tailed *p*-values < 0.05 were considered statistically significant. Statistical analyses were performed using STATA SE 12.0.

## Results

From 1 November 2016 until 1 June 2020, a total of 73 patients with advanced HFREF were implanted with an HM3 LVAD. Of them, 48 were treated according to the old protocol and 25 according to the new protocol. Baseline characteristics and differences between groups are shown in Tab. 1. Mean age was 56 ± 13 years and most patients (78%) were males. Mean left ventricular ejection fraction was 17 ± 7%. The aetiology of HF was ischaemic in 30% of the patients, while the majority had underlying dilated cardiomyopathy (62%). INTERMACS classification was 2–3 in almost 70% of the patients.

**Table 1:** Baseline characteristics of study population according to anticoagulation protocol

Variable	Overall population (*N* = 73)	Old protocol (*n* = 48)	New protocol (*n* = 25)	*P*-value
Age, years	56 ± 13	57 ± 12	53 ± 13	0.27
Female sex (%)	22	17	32	0.13
BMI, kg/m^2^	27 ± 4	27 ± 5	27 ± 3	0.86
Systolic blood pressure, mm Hg	100 ± 14	99 ± 15	102 ± 10	0.48
Diastolic blood pressure, mm Hg	67 ± 12	67 ± 12	67 ± 10	0.90
*Heart failure aetiology*				0.10
– Ischaemic	30	38	16	
– Dilated cardiomyopathy	62	58	72	
– Other	8	4	12	
*INTERMACS class*				0.48
– 2	27	23	36	
– 3	42	46	36	
– 4	27	27	28	
– 5	3	4	0	
*Initial device strategy*				0.028
– BTT	32	29	36	
– BTD	37	29	52	
– DT	32	42	12	
Temporary MCS before LVAD implantation %	10	10	8	0.55
LVEF, %	17 ± 7	15 ± 6	19 ± 7	0.055
PCWP, mm Hg	25 ± 10	25 ± 9	24 ± 11	0.62
mPAP, mm Hg	34 ± 10	34 ± 10	33 ± 11	0.66
CI, l/min per m^2^	1.90 ± 0.41	1.89 ± 0.41	1.92 ± 0.42	0.76
Medical history				
– Hypertension	11	13	8	0.56
– Diabetes mellitus	16	23	4	0.039
– Atrial fibrillation	37	37	36	0.87
– Major myocardial infarction	27	31	20	0.31
– Previous cardiac surgery	23	27	16	0.29
– Cancer	8	10	4	0.34
Serum sodium, mmol/l	138 ± 4	137 ± 5	138 ± 3	0.22
Serum creatinine, mg/dl	1.50 ± 0.93	1.44 ± 0.46	1.62 ± 1.47	0.42
LDH, U/l	232 (186–286)	232 (187–274)	237 (184–288)	0.88
Total bilirubin, μmol/l	17 ± 14	16 ± 12	20 ± 18	0.29
eGFR, mL/min per 1.73 m^2^	59 ± 24	59 ± 25	59 ± 24	1.0
NTproBNP, pg/ml	4473 (2415–6730)	4043 (2415–6486)	5347 (2314–9872)	0.4

Data are %, mean ± standard deviation, or median (interquartile range)

*BMI* body mass index, *BTT* bridge-to-transplant therapy, *BTD* bridge-to-decision therapy, *DT* destination therapy, *MCS* mechanical circulatory support, *LVAD* left ventricular assist device, *LVEF* left ventricular ejection fraction,* PCWP* pulmonary capillary wedge pressure, *mPAP* mean pulmonary artery pressure, *CI* cardiac index before implant, *LDH* lactate dehydrogenase, *eGFR* estimated glomerular filtration rate, *NTproBNP* N-terminal pro-brain natriuretic peptide

Differences in patient characteristics between both groups were small, with numerically more females and significantly less history of diabetes mellitus or angiotensin-converting enzyme inhibitor use in the new protocol group (data not shown). In the new protocol group, a higher percentage of patients received the HM3 LVAD as bridge-to-decision therapy and less often as destination therapy than those in the old protocol group.

### Anticoagulation and INR target levels

**Fig. 1:**
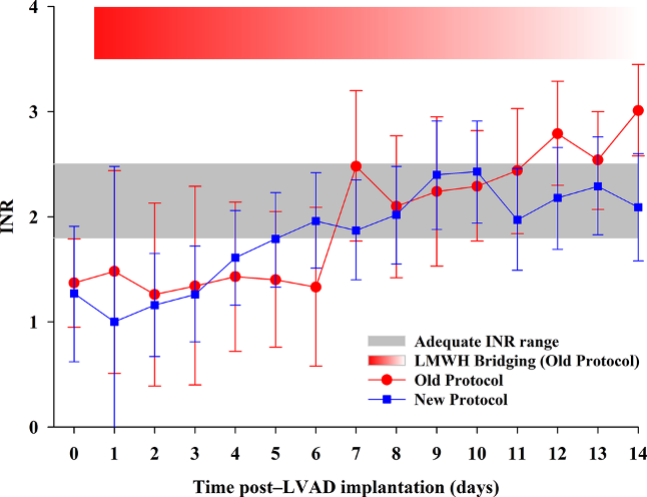
International normalised ratio (*INR*) levels after left ventricular assist device (*LVAD*) implantation in old and new protocol groups. Means and 95% confidence intervals obtained from repeated measures mixed modelling are shown. *P*-value for interaction type of anticoagulation protocol × time is 0.50. *LMWH* low molecular-weight heparin

Fig. 1 shows the results of INR measurements in the first 14 days after LVAD implantation in both protocol groups. In the old protocol group, from day 6, there was a tendency to overshoot the upper limit of the INR range, and this tendency persisted throughout the first 14 days. In the first week, INR levels were below the lower limit of the therapeutic range, but during this time, full-dose LMWH was administered.

In contrast, in the new protocol group, from day 2, there was a gradual increase in INR levels, which reached the therapeutic range around day 5–6. The percentage of patients at each day with an INR measurement within the therapeutic range (INR 1.8–2.5) is shown in Figure S1 (see Electronic Supplementary Material). After 14 days, 72% of the patients in the new protocol group and 46% in the old protocol group had an adequate INR.

### LDH levels as marker of haemolysis

**Fig. 2:**
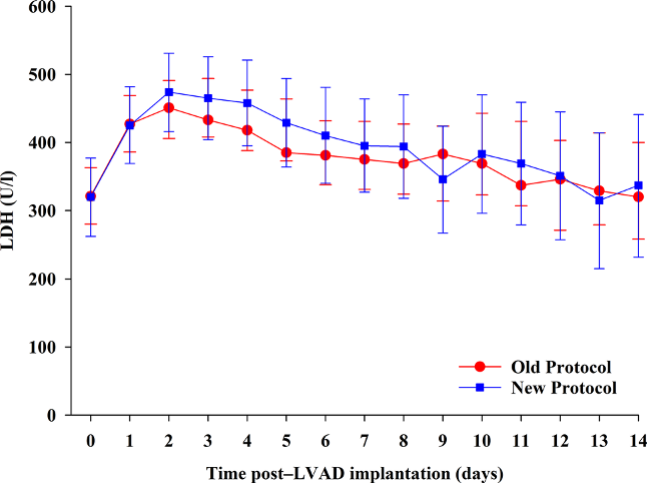
Absolute lactate dehydrogenase (*LDH*) levels over time after left ventricular assist device (*LVAD*) implantation in old and new protocol groups. Means and 95% confidence intervals obtained from repeated measures mixed modelling are shown. *P*-value for interaction type of anticoagulation protocol × time is 0.997

Absolute LDH levels after LVAD implantation over time (Fig. 2) and absolute change in LDH levels from baseline (see Figure S2 in the Electronic Supplementary Material) were evaluated. In both groups, mean LDH levels increased by 130–150 U/l in the first 2 days and slowly decreased to normal levels around 14 days. The mean change in LDH levels in both groups was similar with no statistically significant difference between groups.

### Bleeding events (including tamponade) requiring reoperation

**Fig. 3:**
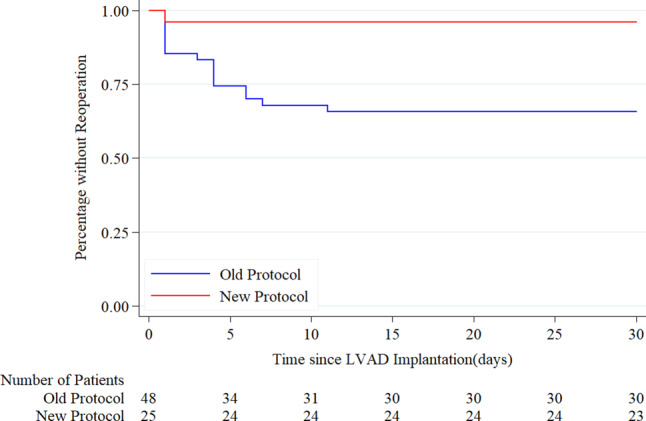
Kaplan-Meier curves for time to first bleeding/tamponade event requiring reoperation after left ventricular assist device (*LVAD*) implantation in old and new protocol groups. *P*-value for log-rank test for difference between anticoagulation protocols is 0.006

Using the old protocol, a total of 19 bleeding/tamponade events requiring reoperation occurred in 16/48 patients, whereas only one event was seen in the new protocol group (*p* = 0.002). When we analysed first events, 33% of the patients in the old protocol group and 4% in the new protocol group required reoperation due to bleeding/tamponade (*p* = 0.005). The number of bleeding/tamponade events requiring reoperation was significantly lower in the new protocol group, as shown by Kaplan-Meier curves for time to first event (Fig. 3).

The hazard ratio (HR) for time to first bleeding/tamponade event requiring reoperation for the new versus old protocol group was 0.10 (95% confidence interval (CI) 0.01–0.79, *p* = 0.029). The excess number of reoperations occurred early after LVAD implantation, suggesting there was no relationship with higher INR levels after 10–14 days in the old protocol group.

### Transfusions

The median number of packed red blood cell transfusions during LVAD implantation was similar for the old and new protocol groups (1 (0–2) vs 1 (0–2), *p* = 0.90). The need for transfusions in the first 3 weeks after LVAD implantation was lower in the new protocol group (0 (0–0), maximum 12 packed red blood cell transfusion) compared with the old protocol group (2 (0–4), maximum 15 packed red blood cell transfusion, *p* = 0.006).

### Length of stay

The median total length of stay after LVAD implantation was 27 days (25–41) in the old protocol, of which 5 days (2–9) were spent in the ICU. In the new protocol group, both median total postoperative length of stay (21 days (19–27), *p* < 0.001) and median length of ICU stay (2 days (2–5), *p* = 0.022) were significantly shorter.

### Thromboembolic events and early postoperative mortality

We observed no thromboembolic events in the early postoperative phase after LVAD implantation in either group up to 1 year of follow-up. There was no suspected or confirmed LVAD pump thrombosis in the entire study population during follow-up.

Early postoperative mortality (first 30 days) was similar in both groups: 3 patients (6.3%) in the old protocol group and 1 patient (5.5%) in the new protocol group (*p* = 0.58). There was no difference in all-cause mortality in the first 6 months after LVAD implantation between the two treatment groups (HR 0.80, 95% CI 0.17–3.85, *p* = 0.78; see Figure S3 in the Electronic Supplementary Material).

## Discussion

In this retrospective study, following a conservative postoperative anticoagulation protocol in patients implanted with the HM3 LVAD was safe and was associated with a lower number of (bleeding) events resulting in reoperation and a shorter duration of stay. This conservative strategy was also not associated with an increased short-term risk of thromboembolic events.

### Long-term thromboembolic events and bleeding complications

With first- and second-generation LVAD devices, there was a substantially increased risk of thromboembolic events due to haemocompatibility issues with the LVAD system being implanted into the blood stream of the HF patient [7, 8]. With the second-generation axial flow devices, the risk of suspected or confirmed LVAD pump thrombosis is 14% at 2 years [3]. Furthermore, the frequency of severe or disabling (embolic) stroke rates is high [3]. Even with the HeartWare Ventricular Assist Device (Medtronic)—a third-generation device—the risk of LVAD reoperation (10%) or stroke (30%) remain high at 2 years [9].

The causes of this increased risk of thromboembolic events in patients implanted with an LVAD are a combination of coagulation cascade activation, including haemolysis due to direct contact of blood (cells) with the rotor of the LVAD, acquired von Willebrand disease impacting thrombocyte function, underlying pro-thrombotic characteristics of HF patients and, immediately after the operation, an increased risk of thromboembolic complications due to the surgical procedure and an associated long rehabilitation period afterwards [10]. This is the reason to advocate a progressive anticoagulation protocol, including vitamin K antagonists together with aspirin.

However, data from the MOMENTUM 3 clinical trial with the third-generation centrifugal flow HM3 LVAD showed a strong reduction in thromboembolic events—with LVAD pump thrombosis occurring in only 1.4% of patients at 2 years—and a reduction in cerebrovascular events compared with the HeartMate 2. [3] Given this low number of thromboembolic events but a bleeding event rate that remained substantial, some studies have evaluated more conservative anticoagulation regimes for HM3 LVAD patients in the chronic phase, mostly in the outpatient setting. For instance, in the small MAGENTUM 1 study, a lower INR threshold (1.5–1.9) was both feasible and not associated with more thromboembolic events [4]. In addition, individual cases have been described in which, mostly due to important bleeding complications, vitamin K antagonists were discontinued without problems in HM3 LVAD patients, even longer term (up to 19 months) [11, 12]. More recently, retrospective analyses from both the MOMENTUM 3 study and the HM3 ELEVATE registry showed no difference in thromboembolic events in patients treated with either low or higher doses of aspirin, nor in bleeding rate [13, 14].

Together, these data suggest that a more conservative approach to long-term anticoagulation in HM3 patients can be safe, although more randomised data are warranted.

### Postoperative complications and anticoagulation

We had observed higher-than-expected bleeding and/or tamponade rates leading to reoperation, which led us to adapt our anticoagulation protocol. As presented herein, in 33% of our first 48 HM3 patients, a reoperation due to excessive bleeding or tamponade was required. Although similar reoperation rates were previously observed within 30 days after LVAD implantation, [15–17] this rate was substantial higher than that in the HM3 CE Mark study and than the overall risk of bleeding requiring surgery in the MOMENTUM 3 study [3, 18]. After changing to our new, conservative protocol, we observed a significant decrease in bleeding and/or tamponade events requiring reoperation to one event (4% of the patients) within the first 30 days.

Even though delaying anticoagulation and antithrombotic therapy might seem risky, our short-term assessments—including change in LDH levels, thromboembolic events and early postoperative mortality—were similar between both protocols. We also did not see any other adverse events related to the slow-start regime of vitamin K antagonist therapy. More importantly, we observed a significant decrease in time spent in the ICU *and* in total length of hospital stay (by almost 1 week) after LVAD implantation, which significantly reduced the in-hospital burden of LVAD patients and resulted in an earlier start of postoperative rehabilitation.

### Limitations

Our study was limited by its retrospective and open design. Our results are a representation of quality improvement of our (postoperative) management of LVAD patients and are not data gathered in a randomised (double-blind) clinical trial. Only the latter can assess causality, which means our results should be interpreted with caution and replicated in a randomised fashion.

There were significantly more patients with a history of diabetes in the old protocol group, and these patients were older and more frequently had a history of cardiac surgery (although both were not statistically different). These factors are likely associated with bleeding risk and may have influenced the results. It could be argued that our old anticoagulation protocol by itself was associated with excess bleeding risk. Even if this was the case, our new protocol shows promising results.

Furthermore, our local protocol specifies an INR range of 1.8 to 2.5 for the HM3 as adequate therapeutic range. Since we observed no (suspected) LVAD pump thrombosis with the HM3 and both anticoagulation protocols had the same INR target, this could not have influenced our results. If any, the lower than generally used INR target would have resulted in a lower bleeding event rate in both groups.

We can also not exclude a ‘learning curve’ while implanting the HM3 LVAD (there were more implantations in the old protocol group), which could have led to better results over time, especially with later implants.

Finally, our results were limited by a small number of LVAD implantations and the study’s single-centre design.

## Conclusion

Our retrospective analysis indicates that a more conservative approach to direct postoperative anticoagulation in patients implanted with an HM3 LVAD is feasible and may be associated with a lower number of bleeding or tamponade events requiring reoperations and less need for transfusion, without evidence of increased risk of thromboembolic events or mortality.

## Supplementary Information

**Supplementary Figure S1 **Percentage of patients in adequate INR range at each timepoint in both protocols

**Supplementary Figure S2** Change in LDH levels from baseline over time after LVAD implantation in both anticoagulation protocol groups

**Supplementary Figure S3** Survival up to 6 months with the old and new anticoagulation protocol
