# Supplementary material for: Conservative initial postoperative anticoagulation strategy after HeartMate 3 left ventricular assist device implantation
Source: Neth Heart J. 2022 Apr 5;30(10):466–72. doi: 10.1007/s12471-022-01671-1 (PMC9475015; doi:10.1007/s12471-022-01671-1)
Supplement: Supplementary file 1 — Supplementary Figure S1 Percentage of patients in adequate INR range at each timepoint in both protocols [file 12471_2022_1671_MOESM1_ESM.docx]

**Supplementary Figure 1. Percentage of patients in adequate INR range at each timepoint in both protocols**

**
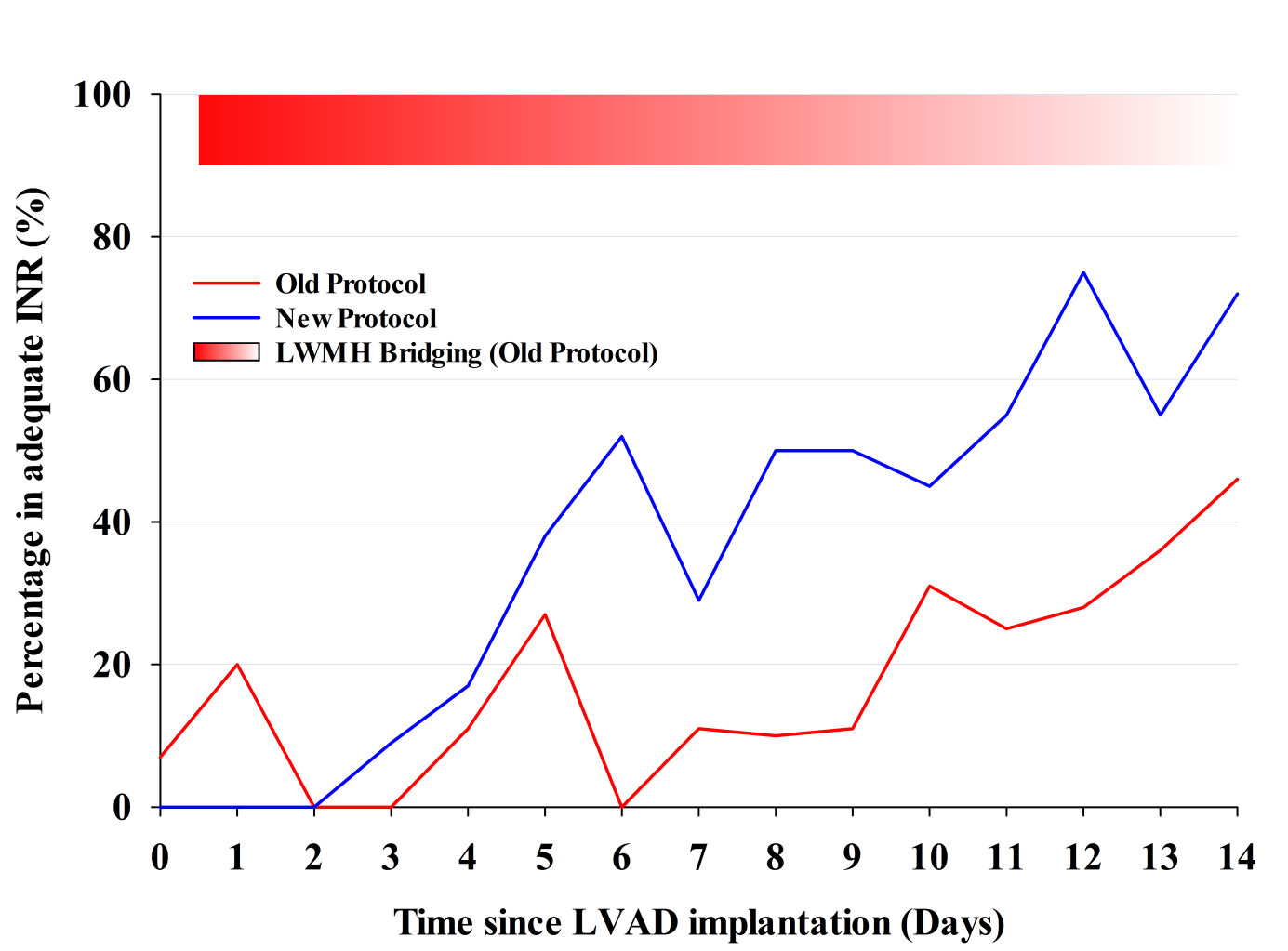
**
