# Supplementary material for: Conservative initial postoperative anticoagulation strategy after HeartMate 3 left ventricular assist device implantation
Source: Neth Heart J. 2022 Apr 5;30(10):466–72. doi: 10.1007/s12471-022-01671-1 (PMC9475015; doi:10.1007/s12471-022-01671-1)
Supplement: Supplementary file 2 — Supplementary Figure S2 Change in LDH levels from baseline over time after LVAD implantation in both anticoagulation protocol groups [file 12471_2022_1671_MOESM2_ESM.docx]

**Supplementary Figure 2. Change in LDH levels from baseline over time after LVAD implantation in both anticoagulation protocol groups**


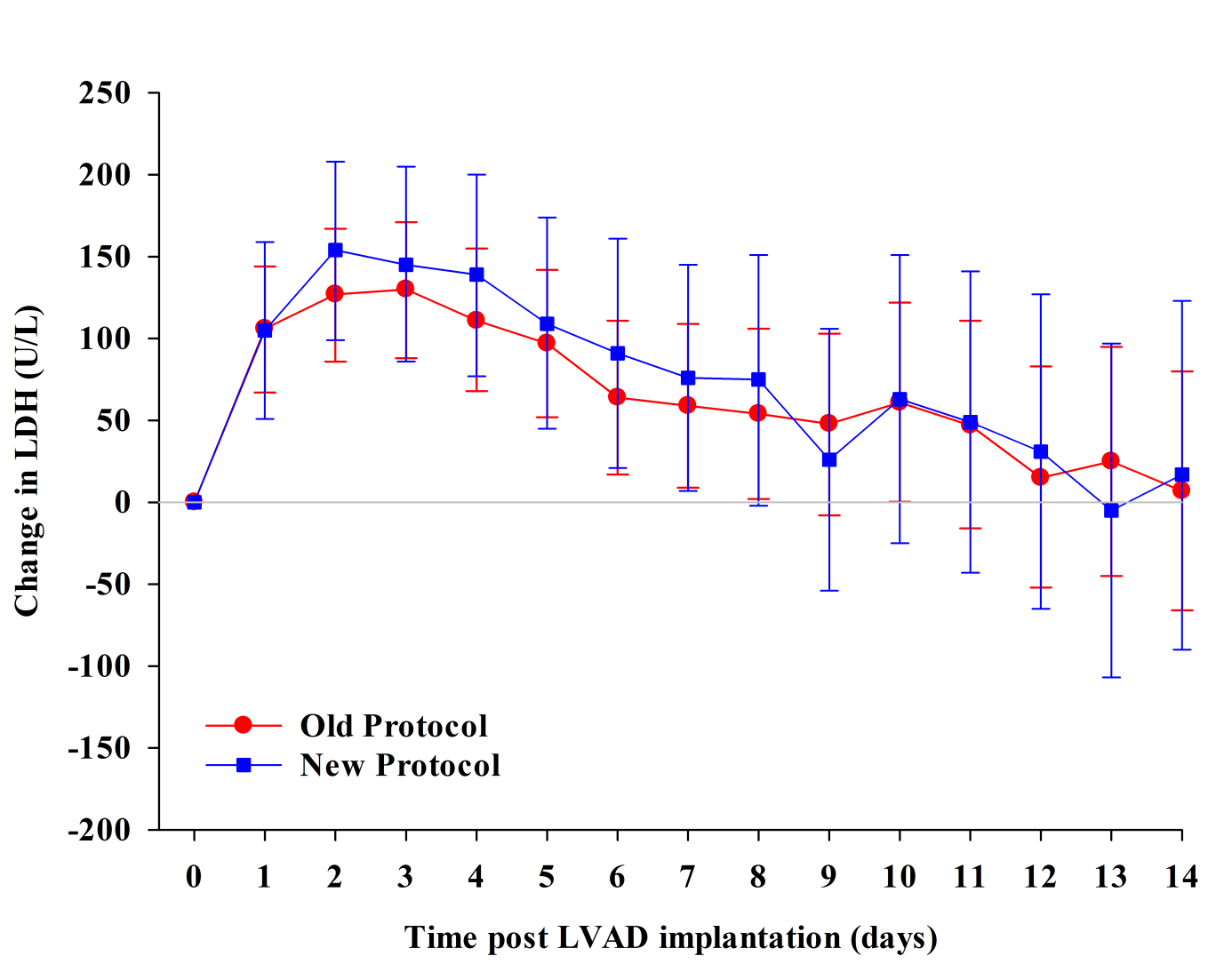
Shown are means and 95% Confidence Intervals obtained from repeated measures mixed modeling.
