# Supplementary figures and images for: Conservative initial postoperative anticoagulation strategy after HeartMate 3 left ventricular assist device implantation
Source: Neth Heart J. 2022 Apr 5;30(10):466–72. doi: 10.1007/s12471-022-01671-1 (PMC9475015; doi:10.1007/s12471-022-01671-1)

**Supplementary Figure 3. Survival up to 6 months with the old and new anticoagulation protocol**


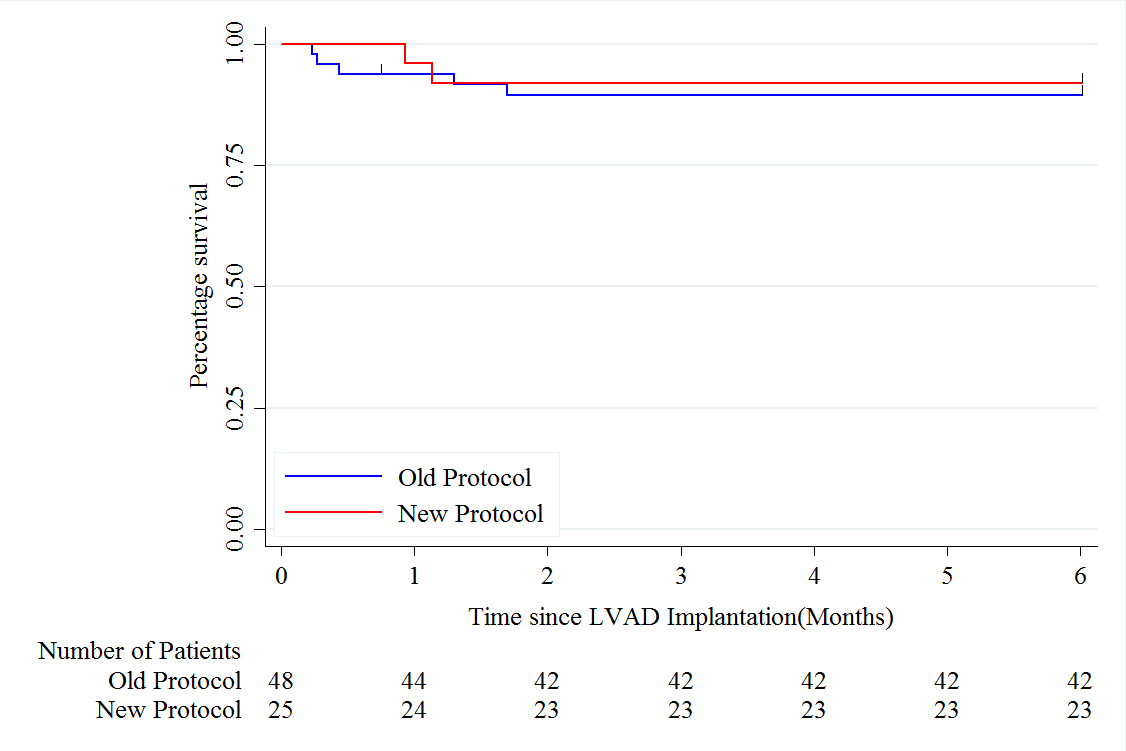

Supplement: Supplementary file 3 — Supplementary Figure S3 Survival up to 6 months with the old and new anticoagulation protocol [file 12471_2022_1671_MOESM3_ESM.docx]
